# Daily and weekly external loads in the microcycle: Characterization and comparison between playing positions on amateur soccer

**DOI:** 10.3389/fspor.2022.943367

**Published:** 2022-09-15

**Authors:** Mauro Miguel, Alberto Cortez, Felix Romero, Nuno Loureiro, Javier García-Rubio, Sergio José Ibáñez

**Affiliations:** ^1^Training Optimization and Sports Performance Research Group (GOERD), Sport Science Faculty, University of Extremadura, Cáceres, Spain; ^2^Sport Sciences School of Rio Maior, Polytechnic Institute of Santarém, Santarém, Portugal; ^3^Life Quality Research Centre (CIEQV), Polytechnic Institute of Santarém, Santarém, Portugal; ^4^Coimbra Business School, Institute of Accounting and Administration of Coimbra (ISCAC), Polytechnic Institute of Coimbra, Coimbra, Portugal

**Keywords:** external load, soccer, daily load, weekly load, playing positions, match reference

## Abstract

Ensuring adequate levels of training and recovery to maximize player performance is critical; however, there are methodological challenges in designing a periodized training program for soccer teams. This study aims to describe and characterize the daily and weekly external load in an amateur soccer team and based on the weighting factors determined by the match reference, compare the external loads between playing positions. Twenty-four amateur soccer players (22.3 ± 1.7 years) were monitored using a global positioning system. Data collected comprises 19 competitive microcycles with a standard structure composed of 3 training sessions (matchday-5, matchday-3, and matchday-2) and one match. Match-reference values were calculated as the mean of the five best values recorded during official matches. The results show, on matchday-5 session, the existence of significant differences between playing positions to relative total distance covered (*p* = 0.050), relative sprint distance (*p* = 0.001), relative moderate-intensity accelerations (*p* < 0.001), relative high-intensity accelerations (*p* = 0.003), relative moderate-intensity decelerations (*p* < 0.001), and relative high-intensity decelerations (*p* = 0.017). On matchday-3 session, there are significant differences to relative very high-speed running distance (*p* = 0.017) and relative moderate-intensity decelerations (*p* = 0.014). On matchday-2 session, there are significant differences to relative high-speed running distance (*p* = 0.025), relative very high-speed running distance (*p* = 0.008), and relative moderate-intensity decelerations (*p* < 0.001). Weekly significant differences are observed between the playing positions to relative moderate-intensity accelerations (*p* = 0.002), relative high-intensity accelerations (*p* < 0.001), and relative moderate-intensity decelerations (*p* < 0.001). The weekly load is characterized by a greater weighting on accelerations and decelerations, compared to distances at very-high speed and sprint. The training loads must respect a standard training model that contemplates the individualization of the physical demands of the match, for each playing position, as for each individual.

## Introduction

Ensuring adequate levels of training and recovery to maximize player performance has continued to drive the necessity to monitor the training load and physical training output of soccer players (Owen et al., 2017); however, designing periodized training programs for team sports athletes poses unique challenges and difficulties (Mujika et al., 2018), and a common problem for coaches is determining the appropriate training loads to prescribe during the week (Clemente et al., 2019a) and the competition phase of the season (Kelly and Counts, 2007). Factors such as the quality of the opposition, the number of training days between matches and any travel associated with playing away matches all influence the between-match periodization of training loads (Kelly and Counts, 2007). Based on this perspective, a periodized approach in the long- and short-term manipulation of training stress and recovery is thought to be essential for the optimal athletic performance and success in competition (Mujika et al., 2018). Therefore, coaches and sports science practitioners should manipulate (Swallow et al., 2021) and monitoring (Ravé et al., 2020) the external training loads to properly periodize their training practices intending to minimize injury risk and optimize players' physical performance. Furthermore, due to the complexity of team sports performance, technical staff in soccer should prescribe daily training load fluctuations during a microcycle that may help to increase or maintain performance throughout the competitive in-season period (Rey et al., 2020).

To achieve this, competitive performance analysis can be used as a reference to apply training load in soccer players (Chena et al., 2021), process in which the periodization components must be simultaneously adapted to meet the desired individual adaptation of each athlete (Boullosa et al., 2020). The great heterogeneity of the team in terms of age, physical conditioning, history of injury, etc. makes it necessary to individualize external training load for each player (Ravé et al., 2020). Since then, gaining knowledge of external training loads relative to the match could be an advantageous strategy, particularly when attempting to optimize position-specific loads (Martín-García et al., 2018), and although a significant time of daily training in team sports is devoted to collective training, individualization of all these aspects would result in a better control of the fitness–fatigue relationship by avoiding any sudden workload spike and thus an increased injury risk (Boullosa et al., 2020). Attending to the external match load differences exhibited, applying a similar “very high-speed running distance” load to full-back and midfielder could potentially lead to overloading the latter position and underloading the former position (Martín-García et al., 2018), which emphasizes the importance of training players as individuals in relation to their positional demands (Owen et al., 2017). Going farther, it can be recommended to collectively and individually program the external training load on a monthly, weekly, and daily level by multiplying the match reference value by a weighting factor (e.g., 3.2 for weekly total distance) (Ravé et al., 2020).

Due to the huge amount of information to be managed with this approach (both collective and individual, as well as multifactorial), the use of big data analysis through the application of the artificial intelligence opens an interesting perspective for predicting injury risk and performance in team sports (Claudino et al., 2019). Recently, some studies (Owen et al., 2017; Stevens et al., 2017; Martín-García et al., 2018; Chena et al., 2021; Modric et al., 2021a) have analyzed the influence of external training loads within weekly training sessions on performance in soccer; however, they all focus on professional contexts, with limited transfer to the amateur context. Thus, and because can be very valuable to express training load data against the match reference (Miguel et al., 2022), since this facilitates the interpretation of the data, and hence the training prescription, this study aims to describe and characterize the daily and weekly external load in an amateur soccer team and based on the weighting factors determined by the match reference, compare the external loads between playing positions. We hypothesized that there would be significant daily and weekly differences in all external load measures between playing positions.

## Materials and methods

### Design

An observational cohort study was carried out in an amateur soccer team throughout the 2018/2019 season in a Portuguese regional competition to determine the incidence of daily and weekly external loads, by playing position. Data collected comprise 19 competitive microcycles with a standard structure composed of three training sessions and a match. Training sessions were classified in relation to the number of days before the next competitive match (Malone et al., 2015b): MD-5 (5 days before match), MD-3 (3 days before match), and MD-2 (2 days before match). For the analysis of the weekly load, the data from the three training sessions were summed.

Given the preliminary nature of our study, for the analysis of training load, only players who fully participated in the three training sessions of the microcycle were included. To analyse the match data, the inclusion criterion was the participation in the entire match. Goalkeeper's data were excluded from the comparative analysis due to the specificity of its playing position, both in training and in the competition.

The specific physical activity profile of individual players during matches is used to plan, according to the physical demands of each player recorded during competitive matches (Martín-García et al., 2018). Consequently, match reference (MRef) values for each GPS parameter allow staff to program external training load at both collective and individual levels (Rago et al., 2020). Collectively, external training load was calculated for each GPS parameter by a collective-weighted factor of MRef values. With the match reference being specific to each player, the calculation of the external training load is individualized (Ingebrigtsen et al., 2015). For each player, external training load was calculated in meters or number of events according to the nature of the GPS parameter (Ravé et al., 2020). For this purpose, MRef values were individually quantified (Akenhead and Nassis, 2016). For determining GPS parameters used to monitor external training load, MRef was arbitrarily calculated as the mean of the five best values recorded during official matches as players were prepared for the most physical demanding matches (Ravé et al., 2020).

### Participants

Twenty-four amateur soccer players (Table 1) belonging to a team that participated in a regional competition (2nd District Division of Santarém) were assessed. The analyzed team played in 1:4:3:3 structure, with two defensive midfielders and one offensive midfielder (these three players are hereinafter referred to as central midfielders). Players were classified according to the playing positions and a total of 132 individual match observations were analyzed: central defender (CD; *n* = 4 players, *n* = 30 cases), fullback (FB; *n* = 4 players, *n* = 30 cases), central midfielder (CM; *n* = 6 players, *n* = 38 cases), wide midfielder (WM; *n* = 5 players, *n* = 24 cases), and forward (F; *n* = 3 players, *n* = 10 cases). Regarding the training sessions, a total of 230 individual observations were analyzed on each training day: central defender (CD; MD-5 = 49 cases, MD-3 = 48 cases, MD-2 = 48 cases), fullback (FB; MD-5 = 30 cases, MD-3 = 35 cases, MD-2 = 34 cases), central midfielder (CM; MD-5 = 67 cases, MD-3 = 66 cases, MD-2 = 67 cases), wide midfielder (W; MD-5 = 52 cases, MD-3 = 52 cases, MD-2 = 50 cases), and forward (F; MD-5 = 32 cases, MD-3 = 29 cases, MD-2 = 31 cases).

**Table 1:** Anthropometric data from the analyzed team.

	**Team**	**Central defenders**	**Fullbacks**	**Central midfielders**	**Wide midfielders**	**Forwards**
Number of players	24*	4	4	6	5	3
Years old	22.3 ± 1.7 y/o	22.5 ± 1.3 y/o	21.3 ± 0.5 y/o	23.3 ± 2.3 y/o	21.2 ± 1.1 y/o	23.0 ± 1.7 y/o
Height	174.5 ± 7.0 cm	178.0 ± 5.9 cm	169.5 ± 4.0 cm	172.3 ± 8.3 cm	178.4 ± 6.9 cm	174.3 ± 6.4 cm
Body Mass	71.1 ± 7.2 kg	75.9 ± 4.7 kg	71.2 ± 6.9 kg	68.0 ± 6.1 kg	69.7 ± 9.1 kg	73.2 ± 9.7 kg
Fat Mass	16.7 ± 3.8%	16.7 ± 6.0%	20.7 ± 2.4%	16.7 ± 1.2%	15.0 ± 2.8%	13.9 ± 3.9%

* “Team” column includes two goalkeepers.

The weekly organization (Morgans et al., 2014; Mujika et al., 2018; Castillo et al., 2021) of the team studied consisted of improving principles and small-principles through small-sided games (SSG) on MD-5 (the initial part of the session was composed of technical and ball possession exercises), improving principles and big-principles through technical-tactical exercises in large spaces on MD-3 (the initial part of the session was composed of technical exercises), and improving the strategic dimension and set pieces through technical–tactical exercises in medium spaces on MD-2 (the initial part of the session was composed of recreational, speed and finishing exercises). The MD-5, MD-3, and MD-2 sessions had an average volume of 100 min (66% of useful time), 94 min (76% of useful time), and 98 min (79% of useful time), respectively. Matches had an average duration of 98 min. To determine the useful time of the training sessions, we excluded instruction times, hydration, breaks between exercise repetitions, transition between exercises and stretching/exercises without displacement.

All players and coaches were informed about the research protocol, requisites, benefits, and risks, and their written consent was obtained before the start of the study. The study protocol was approved by the ethics committee of the local University (n° 67/2017) and performed according to the ethical standards of the Declaration of Helsinki (2013) (World Medical Association, 2013).

### External load quantification

The data from external load were collected using a portable 10 Hz GPS device (PlayerTek, Catapult Innovations, Melbourne, Australia), which also incorporates a tri-axial 100 Hz accelerometer. These types of GPS devices seem to be the most valid and reliable for use in team sports (Scott et al., 2016).

The PlayerTek inertial device was turned on and placed in a specific customized vest pocket located on the posterior side of the upper torso fitted tightly to the body, as is typically used in matches. Both in training and in matches, these devices were turned on 10 min before the start of the warm-up period. During the monitoring period, the GPS devices were placed and checked always by the same coach of the team, and the players always used the same device (Ravé et al., 2020).

The running variables obtained from the GPS were the total distance covered (TDC, m) and the distance covered (m) at three different speed thresholds: “high-speed running distance” (HSRD), 4.0–5.5 m/s, “very high-speed running distance” (VHSRD), 5.5–7.0 m/s, and “sprint distance” (SpD), a speed >7.0 m/s (Miguel et al., 2021). The total number of accelerations and decelerations in two zones were also analyzed: “moderate intensity” (MI Acc./MI Dec.), 2.0–4.0 m/s^2^, and “high intensity” (HI Acc./HI Dec.), >4.0 m/s^2^ (Curtis et al., 2018).

### Statistical analyses

Data are presented as the mean ± standard deviation and in percentage (M ± SD;%) –% relative to the match reference, considering playing positions—the data were relativized considering the MRef as 100% and through which a weighting factor of 1.0 is defined (Ravé et al., 2020). For analysis of variance, all external load measures were relativized (r) based on the MRef of each position. Data normality and homoscedasticity were explored with the Kolmogorov–Smirnoff and Levene tests, showing a non-parametrical distribution. Then, the Kruskal–Wallis test was conducted to analyse, per training day, the differences between the playing positions, and the significance values were adjusted by Bonferroni correction. Effect sizes (ES) were calculated with Hedge's g using absolute values of external load to determine meaningful differences. Magnitudes of difference were classed as trivial, (≤0.2), small (>0.2), moderate (>0.6), large (>1.2), very large (>2.0), and nearly perfect (>4.0) (Hopkins et al., 2009). The level of statistical significance was set at *p* < 0.05. The statistical analyses were performed using IBM SPSS Statistics 28.0 (SPSS Inc., Chicago, IL, USA).

## Results

Descriptive statistics in Table 2 present, by playing position, the match reference, the external load of each training session load (mean ± SD|%, relative to the MRef) and the weekly external load (∑WL, average weekly load, with the sum of the 3 training sessions of the microcycle).

**Table 2:** Daily and weekly descriptive statistics (M ± SD|%, relative to the match reference).

	**Total distance covered, m**					**High-speed running distance, m (4.0–5.5 m/s)**				
	**CD**	**FB**	**CM**	**WM**	**F**	**CD**	**FB**	**CM**	**WM**	**F**
MRef	10,178.1 ± 131.8	11,197.9 ± 339.0	11,962.8 ± 135.2	11,395.3 ± 418.7	11,679.8 ± 254.5	1,262.4 ± 53.4	2,195.6 ± 235.8	2,274.9 ± 60.3	2,117.6 ± 138.6	2,024.0 ± 244.9
MD-5	5,144.6 ± 851.6|51%	5,353.1 ± 693.9|48%	5,676.5 ± 923.2|48%	5,178.6 ± 737.4|46%	5,473.8 ± 897.9|47%	456.7 ± 345.4|36%	581.1 ± 370.6|27%	678.6 ± 432.7|30%	507.0 ± 325.8|24%	597.1 ± 529.7|30%
MD-3	5,905.9 ± 812.3|58%	6,214.2 ± 2,032.0|56%	6,902.0 ± 1,056.7|58%	6,236.9 ± 870.4|55%	6,466.0 ± 895.6|55%	606.1 ± 336.9|48%	812.3 ± 479.8|37%	1,140.1 ± 580.9|50%	845.0 ± 389.5|40%	968.6 ± 608.0|48%
MD-2	4,832.6 ± 508.7|48%	5,286.8 ± 518.1|47%	5,460.8 ± 657.4|46%	5,235.5 ± 498.3|46%	5,204.8 ± 541.6|45%	533.7 ± 254.5|42%	704.6 ± 288.5|32%	803.5 ± 391.0|35%	655.5 ± 244.8|31%	646.5 ± 390.3|32%
∑WL	15,798.3 ± 937.9|155%	17,014.5 ± 1,256.2|152%	17,921.1 ± 1,462.9|150%	16,643.0 ± 1,138.8|146%	17,337.9 ± 1,254.7|148%	1,532.9 ± 859.0|121%	2,122.4 ± 906.5|97%	2,618.5 ± 1,335.8|115%	2,005.9 ± 884.0|95%	2,235.6 ± 1,375.4|111%
	**Very high-speed running distance**, ***m*** **(5.5–7.0 m/s)**					**Sprint distance**, ***m*** **(>7.0 m/s)**				
MRef	398.1 ± 21.1	995.8 ± 70.3	818.5 ± 95.5	767.1 ± 23.2	811.0 ± 70.7	124.6 ± 22.3	399.1 ± 32.4	212.9 ± 34.3	265.4 ± 28.7	369.1 ± 80.9
MD-5	60.9 ± 38.9|15%	110.6 ± 51.3|11%	113.8 ± 59.3|13%	90.8 ± 56.5|12%	113.4 ± 68.2|14%	6.0 ± 15.0|5%	16.0 ± 21.7|4%	7.8 ± 11.7|4%	10.0 ± 11.8|4%	26.9 ± 42.8|7%
MD-3	108.4 ± 68.3|27%	246.9 ± 136.1|25%	228.9 ± 120.0|28%	220.7 ± 101.6|29%	270.9 ± 121.9|33%	14.9 ± 14.2|12%	45.1 ± 43.7|11%	20.7 ± 14.8|10%	31.1 ± 21.1|12%	53.2 ± 56.5|14%
MD-2	170.3 ± 58.1|43%	286.6 ± 103.4|29%	278.1 ± 98.1|34%	269.3 ± 80.5|35%	273.3 ± 101.4|34%	29.5 ± 24.6|24%	61.0 ± 46.7|15%	45.7 ± 38.6|22%	52.8 ± 43.8|20%	104.8 ± 110.4|27%
∑WL	337.0 ± 99.7|85%	664.3 ± 195.0|67%	603.7 ± 203.5|74%	578.9 ± 177.3|76%	654.5 ± 196.4|81%	51.5 ± 29.8|41%	135.0 ± 91.7|34%	69.4 ± 45.5|33%	96.3 ± 58.2|36%	173.2 ± 136.6|47%
	**Moderate intensity accelerations**, ***n*** **(2.0–4.0 m/s**^**2**^**)**					**High intensity accelerations**, ***n*** **(>4.0 m/s**^**2**^**)**				
MRef	213 ± 12	292 ± 12	290 ± 13	277 ± 23	266 ± 23	24 ± 2	44 ± 1	43 ± 2	45 ± 3	40 ± 6
MD-5	167 ± 40|78%	186 ± 39|64%	191 ± 45|66%	169 ± 35|61%	188 ± 45|71%	18 ± 6|75%	24 ± 8|55%	24 ± 11|56%	26 ± 8|58%	24 ± 8|60%
MD-3	146 ± 41|69%	169 ± 67|58%	186 ± 47|64%	162 ± 33|59%	176 ± 37|66%	15 ± 5|63%	25 ± 10|57%	19 ± 7|44%	25 ± 9|56%	21 ± 5|53%
MD-2	90 ± 18|42%	113 ± 22|39%	117 ± 26|40%	106 ± 23|38%	111 ± 24|42%	19 ± 7|79%	27 ± 7|61%	27 ± 7|63%	30 ± 7|67%	27 ± 7|68%
∑WL	394 ± 56|185%	468 ± 56|160%	494 ± 72|170%	439 ± 48|159%	478 ± 65|180%	50 ± 10|208%	76 ± 14|173%	68 ± 14|158%	79 ± 16|176%	72 ± 12|180%
	**Moderate intensity decelerations**, ***n*** **(−2.0 to** **−4.0 m/s**^**2**^**)**					**High intensity decelerations**, ***n*** **(** **>** **−4.0 m/s**^**2**^**)**				
MRef	169 ± 3	245 ± 9	279 ± 40	236 ± 16	223 ± 12	32 ± 3	51 ± 4	58 ± 5	58 ± 7	56 ± 2
MD-5	161 ± 36|95%	177 ± 34|72%	186 ± 37|67%	165 ± 35|70%	182 ± 38|82%	19 ± 9|59%	23 ± 10|45%	26 ± 8|45%	23 ± 7|40%	23 ± 10|41%
MD-3	129 ± 38|76%	149 ± 62|61%	172 ± 43|62%	144 ± 32|61%	154 ± 35|69%	17 ± 7|53%	23 ± 12|45%	23 ± 7|40%	23 ± 9|40%	25 ± 7|45%
MD-2	88 ± 15|52%	110 ± 21|45%	118 ± 22|42%	107 ± 17|45%	108 ± 25|48%	14 ± 6|44%	20 ± 9|39%	20 ± 8|35%	22 ± 8|38%	22 ± 10|39%
∑WL	370 ± 51|219%	431 ± 52|176%	475 ± 58|170%	416 ± 45|176%	446 ± 52|200%	48 ± 16|150%	64 ± 17|126%	65 ± 16|112%	68 ± 16|117%	70 ± 17|125%

MRef, match reference, arbitrarily calculated as the mean of the five best values recorded during official matches; MD-5, five days before match-day; MD-3, three days before match-day; MD-2, two days before match-day; ∑WL, average weekly load, with the sum of the 3 training sessions of the microcycle; CD, central defenders; FB, Fullbacks; CM, central midfielders; WM, wide midfielders; F, forwards.

### External load comparison between playing positions on MD-5

**Figure 1:**
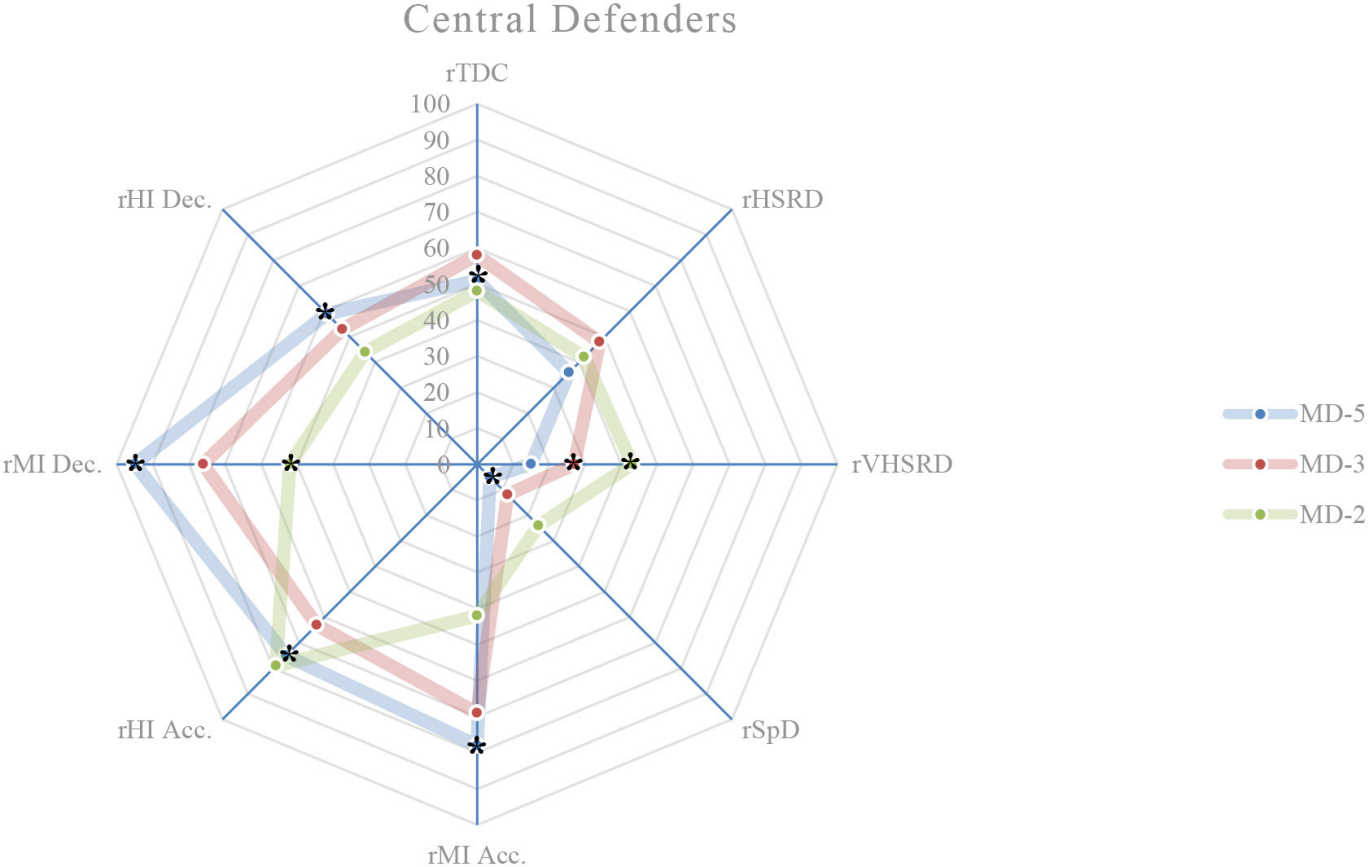
Daily external load (based on MRef) of central defenders. *Location of the significant differences, *p* < 0.05.

**Figure 2:**
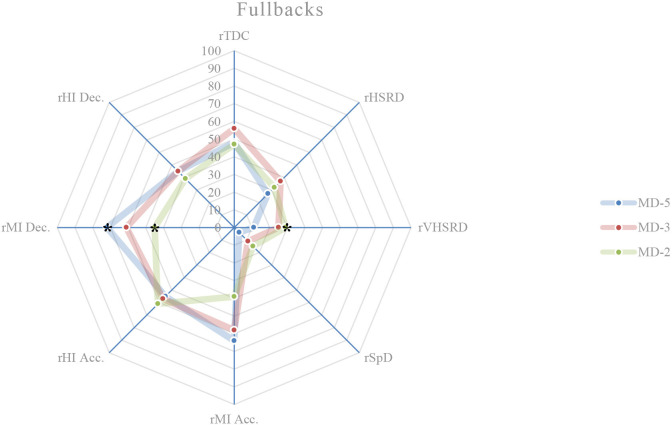
Daily external load (based on MRef) of fullbacks. *Location of the significant differences, *p* < 0.05.

**Figure 3:**
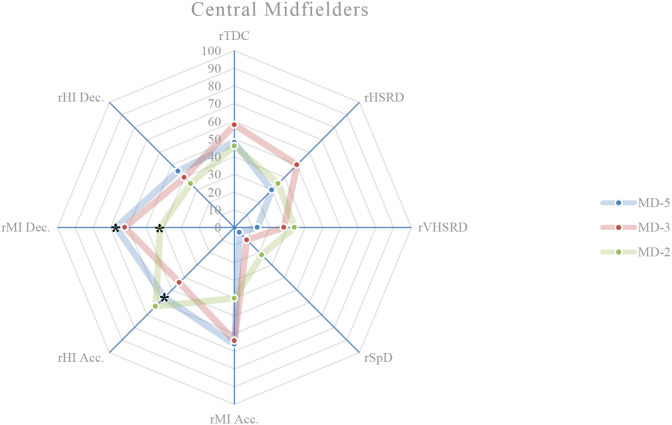
Daily external load (based on MRef) of central midfielders. *Location of the significant differences, *p* < 0.05.

**Figure 4:**
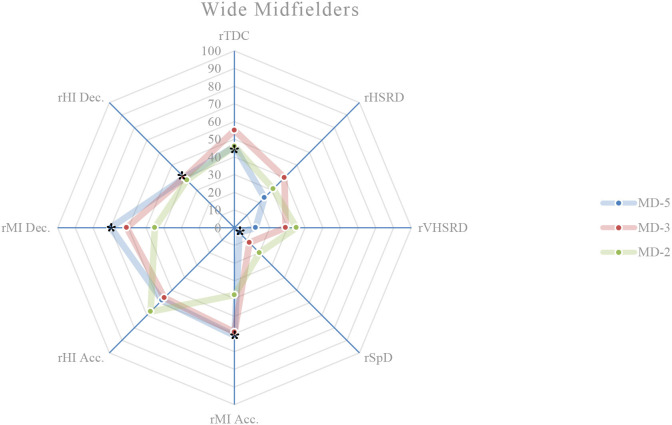
Daily external load (based on MRef) of wide midfielders. *Location of the significant differences, *p* < 0.05.

**Figure 5:**
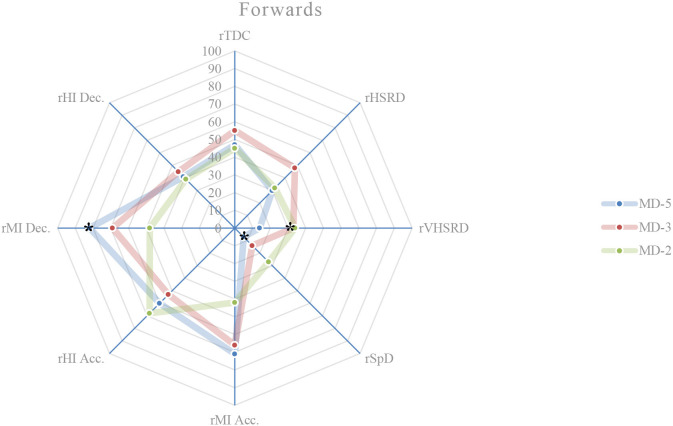
Daily external load (based on MRef) of forwards. *Location of the significant differences, *p* < 0.05.

On this training day, the results show the existence of significant differences between playing positions relatively to rTDC (*p* = 0.050), rSpD (*p* = 0.001), rMI Acc. (*p* < 0.001), rHI Acc. (*p* = 0.003), rMI Dec. (*p* < 0.001), and rHI Dec. (*p* = 0.017). A more detailed analysis shows that: central defenders exhibit a higher rTDC compared to wide midfielders (*p* = 0.027; ES = 0.59) (Figures 1, 4); central defenders exhibit a lower rSpD compared to fullbacks (*p* = 0.001; ES = 0.06) (Figures 1, 2), wide midfielders (*p* = 0.045; ES = 0.01) and forwards (*p* = 0.002; ES = 0.14) (Figures 4, 5); central defenders exhibit a higher rMI Acc. compared to wide midfielders (*p* = 0.001; ES = 0.82) (Figures 1, 4); central defenders exhibit a higher rHI Acc. compared to central midfielders (*p* = 0.001; ES = 0.68) (Figures 1, 3); central defenders exhibit a higher rMI Dec. compared to fullbacks (*p* = 0.011; ES = 0.84) (Figures 1, 2); central midfielders (*p* = 0.000; ES = 1.12) and wide midfielders (*p* = 0.000; ES = 0.99) (Figures 3, 4); forwards exhibit a higher rMI Dec. compared to central midfielders (*p* = 0.034; ES = 0.76) (Figures 3, 5); central defenders exhibit a higher rHI Dec. compared to wide midfielders (*p* = 0.011; ES = 0.70) (Figures 1, 4).

### External load comparison between playing positions on MD-3

On this training day, the results show the existence of significant differences between playing positions relatively to rVHSRD (*p* = 0.017) and rMI Dec. (*p* = 0.014). A more detailed analysis shows that: forwards exhibit a higher rVHSRD compared to central defenders (*p* = 0.009; ES = 0.37) (Figures 1, 5); and, based on Bonferroni correction, there is no evidence of a difference between the pairs in rMI Dec.

### External load comparison between playing positions on MD-2

On this training day, the results show the existence of significant differences between playing positions relatively to rHSRD (*p* = 0.025), rVHSRD (*p* = 0.008), and rMI Dec. (*p* < 0.001). A more detailed analysis shows that: based on Bonferroni correction, there is no evidence of a difference between the pairs in rHSRD; central defenders exhibit a higher rVHSRD compared to fullbacks (*p* = 0.007; ES = 0.81) (Figures 1, 2); central defenders exhibit a higher rMI Dec. compared to fullbacks (*p* = 0.025; ES = 0.71) (Figures 1, 2), and central midfielders (*p* = 0.000; ES = 0.87) (Figure 3).

### Weekly external load comparison between playing positions

**Figure 6:**
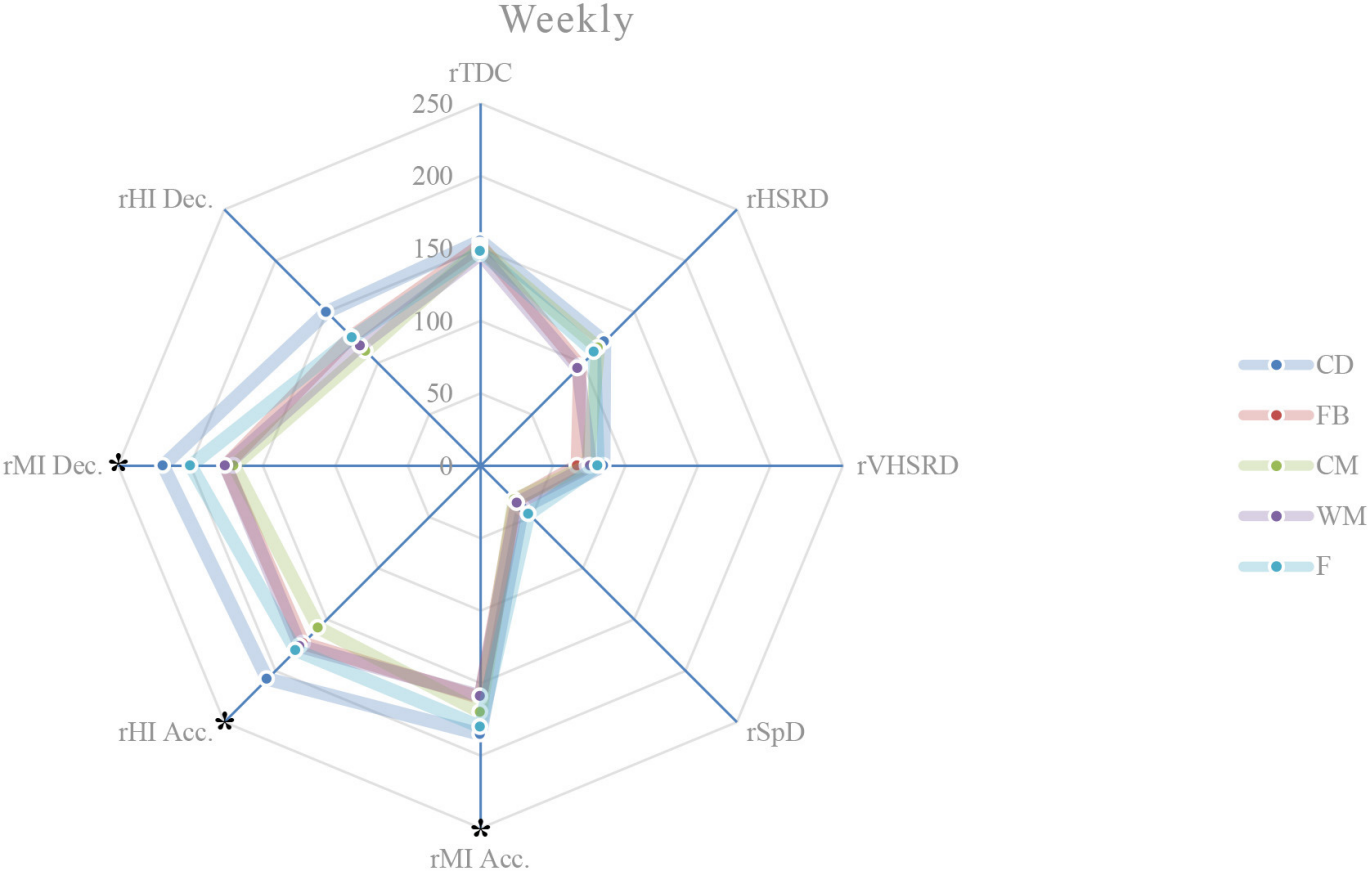
Weekly external load (based on MRef) of all playing positions. *Location of the significant differences, *p* < 0.05.

Weekly, significant differences are observed between the playing positions in relation to the rMI Acc. (*p* = 0.002), rHI Acc. (*p* < 0.001), and rMI Dec. (*p* < 0.001) (Figure 6). A more detailed analysis shows that: central defenders exhibit a higher rMI Acc. compared to wide midfielders (*p* = 0.010; ES = 1.19), and fullbacks (*p* = 0.026; ES = 1.07); central defenders exhibit a higher rHI Acc. compared to fullbacks (*p* = 0.031; ES = 0.94), and central midfielders (*p* = 0.000; ES = 1.36); central defenders exhibit a higher rMI Dec. compared to central midfielders (*p* = 0.000; ES = 1.89), fullbacks (*p* = 0.000; ES = 1.64), and wide midfielders (*p* = 0.000; ES = 1.68); forwards exhibit a higher rMI Dec. compared to central midfielders (*p* = 0.009; ES = 1.35).

## Discussion

This study aims to describe and characterize the daily and weekly external load in an amateur soccer team and based on the weighting factors determined by the match reference, compare the external loads between playing positions. While the description of the daily and weekly external load, presented in Table 2 and in Figures 1–6, allows the characterization of the work performed, the analysis of variance between playing positions shows significant findings.

### Weekly characterization of the daily and weekly external load

Of the three weekly training sessions (Table 2), the MD-3 session presented the highest values of rTDC (0.55–0.58) and rHSRD (0.37–0.50), having the MD-2 and MD-5 sessions identical values. In relation to rVHSRD and rSpD, the MD-5 session presented the lowest values (0.11–0.15, and 0.04–0.07, respectively), increasing their incidence with the proximity of competition. About the rMI Acc. and rMI Dec., these load measures presented on MD-5 the highest values (0.61–0.78, and 0.55–0.75, respectively), decreasing their incidence with the proximity of competition. Regarding the rHI Acc., the MD-2 session presented the highest values and the MD-3 the lowest (0.61–0.79, and 0.44–0.63, respectively)—for the fullbacks the MD-5 session was the one with the lowest values (0.55). As for rHI Dec., the MD-2 session presented the lowest values (0.35–0.44)—MD-5 and MD-3 present identical demands (0.40–0.59).

Among several studies (Malone et al., 2015a; Owen et al., 2017; Stevens et al., 2017; Martín-García et al., 2018; Clemente et al., 2019a; Sanchez-Sanchez et al., 2019; Kelly et al., 2020; Oliveira et al., 2020; Chena et al., 2021; Swallow et al., 2021), there is a tendency that the closer proximity to the match day results in a predisposition to decrease the training load, particularly from the middle of the week until MD-1, highlighting a conscious tapering period (Clemente et al., 2019a); however, all of them have particularities. Boullosa et al. (2020) claim that these different loading patterns are also mirrored in tapering strategies, however, clarifies that the reduced loads on the days before matches in team sports cannot be considered as tapering. In this sense, Saidi et al. (2019) report that weekly practices mostly reflect an attempt to recover sufficiently from matches. While in some studies, the decrease in load near competition day comprises all the metrics, the results of our study show two distinct trends. For us, it is difficult to compare the results with other studies, because although there are common training days (e.g., MD-2), none of them present a schedule equal to that of this investigation, and according to Teixeira et al. (2021), the training load variation seems to be influenced, among other factors, by the type of weekly schedule, as well as the type of weekly microcycle (i.e., one-, two-, and three-match week) appears to decisively influence the load distribution. However, we consider that with one-match week, and with a completely stable weekly schedule, the combination of technical, technical-tactical and physical work, together with: the use of large spaces in the exercises performed on MD-3 session resulted in the existence of higher values of rTDC and rHSRD on this training day; the use of reduced spaces on MD-5 session mitigates the existence of rVHSRD and rSpD, on this training day, of this type of physical demand, with higher values being observed when performing analytical exercises, specific to achieving very high speeds (on MD-2); the use of reduced spaces resulted in higher values of rMI Acc. and rMI Dec. on MD-5, however without the high intensity accelerations and decelerations presenting the greatest demand in this session. Finally, the values of rHI Acc. and rHI Dec. do not present identical distribution in the three training sessions—the speed training on MD-2 makes this day also where the most HI Acc. are performed. Grünbichler et al. (2020), based on an microcycle composed of 5 training sessions, MD-5 to MD-1, suggest that for an optimal pre-match preparation, coaches should plan sprint (>7.0m/s) training session during the week (3–4 days before the match) and additionally should avoid excessive training loads and long sessions 1 day before the match, while Modric et al. (2021a), with the same type of weekly schedule, indicate that players should be exposed to a minimum of 75–80% of the high-intensity running (>5.5 m/s) normally characterizing a match in the middle of the week, as well as training methodology that utilizes a “high-volume and low-intensity” training session in the second day after the match (i.e., on MD-5) may positively impact success in soccer. Although there are trends and similarities in the management of daily loads during the microcycle, this management must be unique, situational, and evolutionary to meet the needs of the team and the players, as a group and individuality.

Weekly (in the set of 3 training sessions) found values between 1.46 and 1.55 (weighting factors) for rTDC; 0.95 and 1.21 for rHSRD; 0.67 and 0.85 for rVHSRD; 0.33 and 0.47 for rSpD; 1.60 and 1.85 for MI Acc.; 1.58 and 2.08 for HI Acc.; 1.70 and 2.19 for MI Dec.; 1.12 and 1.50 for HI Dec. Clemente et al. (2019b), in weeks with three training sessions, report 1.8 for rTDC, 1.2 for running distance, 1.1 for high-speed running distance, 2.2 for high accelerations (>3 m/s^2^) and 1.6 for high decelerations (> −3 m/s^2^). In weeks with five training sessions, weighting factors of 3.5, 2.3, 2.3, 4.1, and 3.4 are reported, respectively. Modric et al. (2021b) describe values between 1.74 and 2.05 for rTDC, 0.63 and 1.30 for high-intensity distance (>5.5 m/s), and of 2.01 for high-intensity accelerations (>3.0 m/s^2^) and 1.47 for high-intensity decelerations (<-3.0 m/s^2^). Sanchez-Sanchez et al. (2019) revealed a value of 2.90 for rTDC, 2.10 for high-intensity distance (4.0–5.5 m/s), 1.90 for sprinting distance (>5.5 m/s), 3.00 for accelerations (>2.5 m/2^2^) and 3.00 for decelerations (<-2.5 m/s^2^). Stevens et al. (2017) found that, relative to match values, acceleration load during training was in general higher (3.10–3.90) than total distance ran (3.10) and distance ran at high speeds (2.10). Chena et al. (2021) found values of 2.77 for rTDC, 2.00 for high-speed running (>5.83 m/s), 2.30 for accelerations (>2.5 m/s^2^), and 2.41 for decelerations (<-2.5 m/s^2^). However, these studies present a different number of training sessions (Sanchez-Sanchez et al., 2019; Modric et al., 2021b) or add the competition load to the weekly data (Stevens et al., 2017; Chena et al., 2021).

Unexpectedly, we found that the amount of work done in the week relative to the rVHSRD and rSpD never reaches the MRef value, while the rTDC presents one-and-a-half times the MRef value. Also Modric et al. (2021b) report that weekly high-intensity distance covered (>5.5 m/s), for some playing positions, was lower compared to the match values. Regarding the load measures associated with accelerations and decelerations, we concluded that they are the most requested, reaching the point of doubling the MRef value. These results are in agreement with Clemente et al. (2019a) and Modric et al. (2021b), who demonstrated that through weekly training sessions, the TDC and accelerations/decelerations were more emphasized than the high-intensity distance covered (e.g., VHSRD and SpD). According to Modric et al. (2021a), training approaches usually contain drills that are performed in small areas, players are limited in reaching higher running speeds. As a consequence of not being exposed to high intensity running patterns, players mostly do not meet the loads imposed during matches. This is reinforced by Santos et al. (2021), who recommend that smaller formats seem to promote higher exercise intensity but may be a limitation for the occurrence of higher running speeds, so it would be recommended to increase pitch size if coaches want to design tasks with greater focus on speed. Casamichana et al. (2018) conclude by explaining that those who wish to work on high-speed movements should design SSGs on larger pitches, giving priority to length rather than width for the same playing surface. Therefore, we alert to the importance of attending to exercises that require higher running speeds, for which the definition of suitable playing areas (large, particularly in their length) is crucial. Alternatively, the use of analytical exercises (without the ball) may complement the requirements of the training session when they consciously do not meet these physical objectives. Additionally, Clemente et al. (2019b), emphasizing the importance of the number of weekly training sessions, refer that to achieve a rTDC weighting factor of 2.0 (as an example) only three sessions/week are necessary, but in the case of high-speed running (5.55–6.95m/s), 4 sessions/week are necessary. This analysis anticipates that coaches should be very careful when designing their plans, in order to compensate for the reduced training time involved in the sum of the week, which limits the achievement of the desired weighting factors.

Boullosa et al. (2020) affirm that in team sports there is no “true peaking” at any time of the season, but a performance plateau on the level of physical and physiological adaptations that allows appropriate technical-tactical performances over time, and considering the periodization of intra-weekly and weekly loads, Kelly et al. (2020) describe that there are methodological challenges inherent in soccer, which limit the ability to determine the direct influence of training load on team match physical performance and/or success and therefore our understanding of what may constitute optimal periodization of training. Consequently, we recommend that the training workloads, most often collectively performed, with predefined daily objectives included in the weekly microcycle (Boullosa et al., 2020), respect a standard training model, which contemplates specific physical demands of the game. As found by Guerrero-Calderón et al. (2021), both the total distance, high-intensity running distance and sprint distance, covered in match by players showed strong relationships to training load realized during the previous week, which highlights the importance of the training intensity to be high, while the volume must be kept low in order to achieve an increased physical output in the next match. Lastly, the stabilization of the training workloads will prevent excessive fatigue and decreased performance (Chena et al., 2021).

### Comparison of the daily and weekly external load between playing positions

The results of our investigation show that the daily and weekly external load that players are subjected to is not identical between all playing positions, which is in accordance with results of Modric et al. (2021b). Concurrently, Akenhead et al. (2016) describe that the observed interposition differences in external load variables were smaller than those frequently reported within the literature for competitive matches.

On MD-5 session, it is observed that the central defenders differ significantly from the other playing positions, and in several load measures. Except for rSpD, where they have a significantly lower load than the fullbacks (trivial ES), wide midfielders (trivial ES) and forwards (trivial ES), the central defenders exhibit significantly higher loads in terms of rTDC (small ES compared to wide midfielders), rMI Acc. (moderate ES compared to wide midfielders), rHI Acc. (moderate ES compared to central midfielders), and rMI Dec. (moderate ES compared to central midfielders, wide midfielders, and forwards). On MD-3 session, central defenders have a significantly lower rVHSRD when compared to forwards (small ES). On MD-2 session, central defenders differ significantly from fullbacks (rVHSRD, moderate ES; rMI Dec., moderate ES) and from central midfielders (rMI Dec., moderate ES), presenting higher values for any of these load measures. In the weekly load, the central defenders differ significantly from the fullbacks (rMI Acc., moderate ES; rHI Acc., moderate ES; rMI Dec., large ES), central midfielders (rHI Acc., large ES; rMI Dec., large ES), and from wide midfielders (rMI Acc., moderate ES; rHI Dec., large ES), presenting higher values in all these measures. Finally, the forwards differ significantly from the central midfielders, presenting a higher rMI Dec. (large ES), which is relevant, since no significant differences between these two playing positions are identified on a daily basis.

Castillo et al. (2021) state that almost all measures' external loads were higher during matches when compared with training sessions. Malone et al. (2015a) reported limited positional differences in the sessions leading up to the match, while Martín-García et al. (2018) found that the external load of the microcycle varied substantially based on the players tactical role in the team. Our results show that the central defenders are the playing position that most differs from the others, particularly on MD-5, where they tended to present, for each measure of external load, lower absolute values than the other playing positions; however significantly higher if relativized to the MRef. The use of SSG on this training day, some of them without a defined structure, seems to normalize the external load and therefore, not clearly individualize the load to the playing position. Castillo et al. (2021) recommend that technical staff should approach their training tasks in each specific training session to get the conditional objectives in terms of neuromuscular demands (e.g., high-intensity accelerations), endurance components (e.g., total distance covered), and speed actions (e.g., distance covered at above 7.0 m/s). These analysis corroborates the statement by Boullosa et al. (2020) consider that the individualization is the key in a multifactorial periodization model because it allows a more flexible approach on a daily basis, and defines that the major advantage of this individualized approach is that it avoids any excessive loading, and therefore sudden individual workload spikes. Although in team sports, the training loads are most often collectively performed, with predefined daily objectives included in the weekly microcycle (Mujika et al., 2018), the need to adjust planning to an individual approach becomes evident, assuming the training a coherent demand with the requirements of the match. Many studies, ours included, have referred to the individualization of loads, taking into account the position occupied by the players. This is reinforced by Nobari et al. (2022) who suggest that coaches should be aware of the specific external load demands to prescribe more representative training tasks for each positional role. However, it is necessary to go even further and meet the needs not only of each playing position but also of each player. Therefore, to meet the needs of each player, a weekly and a daily factors must be individualized, either to increase or to reduce the external training load with respect to the collective external training load (Ravé et al., 2020). By collecting data from individual players throughout the season, coaches can predict what the load pattern will be in the match (Guerrero-Calderón et al., 2021).

Although this investigation provides valuable results and analyses on this research topic, there are some limitations to consider: firstly, the main limitation of this study is the fact that only one team was observed, which is a very common obstacle in studies with soccer players (Clemente et al., 2019b); secondly, we did not consider the time of participation in the previous match in the analysis of the daily and weekly load; finally, in longitudinal data, there are two major dimensions to consider, individual and time; however, we have grouped the entire analysis into a single and comprehensive period, the competitive period.

## Conclusions

Based on MRef, the present study analyses the daily and weekly external loads by playing position. Different patterns are observed regarding the external load measures evaluated: While rTDC and rHSRD have a peak incidence in MD-3, rVHSRD and rSpD increasing their incidence with the proximity of competition. In contrast, rMI Acc. and rMI Dec. decreasing their incidence with the proximity of competition. In relation to rHI Acc. and rHI Dec., the trend is opposite, while the rHI Acc. presents higher values in MD-2, on this training day the rHI Dec. presents the lowest values. Having been observed daily differences in the external load, these mainly consist of accelerations and decelerations, as well as distances covered at very high speeds (interestingly, the rTDC appears to be very homogeneous between the different playing positions). Concomitantly, the weekly load is characterized by a greater weighting on accelerations and decelerations, compared to distances at very high speed and sprint (surprisingly, the rVHSRD and rSpD never reach 100% of the effort required by the game, measuring values below 50% for rSpD).

Future studies should be able to prescribe rigorous methodologies and training exercises that make it possible to adapt the physical demands of training, to those required by the game, for each position and individual.

## Practical applications

- The training loads must respect a methodology and a standard training model that contemplates the individualization of the physical demands of the match, for each playing position, as for each individual.
- The distribution of daily loads must be carefully planned, matching the dominant (technical-tactical) to the regime (physical) of the training session. The methodology and periodization used should guide the daily regimen, where the use of analytical exercises (without the ball) can complement the requirements of the training session when they consciously do not meet these physical objectives.
- Assuming that training requirements should be coherent with the demands of the match, it will be essential to attend to exercises that require higher running speeds, and for which the definition of suitable playing areas is crucial.
- It becomes essential to map the “physical costs” of each training exercise, to ensure an effective control of the daily load. Thus, when planning training, it is possible to predict/anticipate the external load that players will be subjected to and identify compensation needs.

## Data availability statement

The original contributions presented in the study are included in the article/supplementary material, further inquiries can be directed to the corresponding author.

## Ethics statement

Study protocol was approved by the Ethics Committee of the University of Extremadura (n° 67/2017). The patients/participants provided their written informed consent to participate in this study.

## Author contributions

Conceptualization and methodology: MM and NL. Validation and writing—review and editing: JG-R and SI. Formal analysis and data curation: MM, AC, and FR. Investigation and writing—original draft preparation: MM. Resources: NL. Visualization: JG-R. Supervision and funding acquisition: SI. Project administration: MM, NL, JG-R, and SI. All authors have read and agreed to the published version of the manuscript.

## Funding

This study has been partially subsidized by the Aid for Research Groups (GR21149) from the Regional Government of Extremadura (Department of Economy, Science and Digital Agenda), with a contribution from the European Union from the European Funds for Regional Development, and by the Portuguese Foundation for Science and Technology, I.P., Grant No. UIDP/04748/2020.

## Conflict of interest

The authors declare that the research was conducted in the absence of any commercial or financial relationships that could be construed as a potential conflict of interest.

## Publisher's note

All claims expressed in this article are solely those of the authors and do not necessarily represent those of their affiliated organizations, or those of the publisher, the editors and the reviewers. Any product that may be evaluated in this article, or claim that may be made by its manufacturer, is not guaranteed or endorsed by the publisher.
